# Acute stress induces an aberrant increase of presynaptic release of glutamate and cellular activation in the hippocampus of BDNF^Val/Met^ mice

**DOI:** 10.1002/jcp.30833

**Published:** 2022-07-31

**Authors:** Laura Musazzi, Paolo Tornese, Nathalie Sala, Francis S. Lee, Maurizio Popoli, Alessandro Ieraci

**Affiliations:** ^1^ Department of Medicine and Surgery University of Milano‐Bicocca Monza Italy; ^2^ Dipartimento di Scienze Farmaceutiche University of Milan Milan Italy; ^3^ Department of Psychiatry Weill Cornell Medical College New York New York USA

**Keywords:** acute stress, BDNF Val66Met polymorphism, gene expression, glutamate release, mood disorders, synaptic mechanisms

## Abstract

Stressful life events are considered major risk factors for the development of several psychiatric disorders, though people differentially cope with stress. The reasons for this are still largely unknown but could be accounted for by individual genetic variants, previous life events, or the kind of stressors. The human brain‐derived neurotrophic factor (BDNF) Val66Met variant, which was found to impair intracellular trafficking and activity‐dependent secretion of BDNF, has been associated with increased susceptibility to develop several neuropsychiatric disorders, although there is still some controversial evidence. On the other hand, acute stress has been consistently demonstrated to promote the release of glutamate in cortico‐limbic regions and altered glutamatergic transmission has been reported in psychiatric disorders. However, it is not known if the BDNF Val66Met single‐nucleotide polymorphism (SNP) affects the stress‐induced presynaptic glutamate release. In this study, we exposed adult male BDNF^Val/Val^ and BDNF^Val/Met^ knock‐in mice to 30 min of acute restraint stress. Plasma corticosterone levels, glutamate release, protein, and gene expression in the hippocampus were analyzed immediately after the end of the stress session. Acute restraint stress similarly increased plasma corticosterone levels and nuclear glucocorticoid receptor levels and phosphorylation in both BDNF^Val/Val^ and BDNF^Val/Met^ mice. However, acute restraint stress induced higher increases in hippocampal presynaptic release of glutamate, phosphorylation of cAMP‐response element binding protein (CREB), and levels of the immediate early gene c‐fos of BDNF^Val/Met^ compared to BFNF^Val/Val^ mice. Moreover, acute restraint stress selectively increased phosphorylation levels of synapsin I at Ser^9^ and at Ser^603^ in BDNF^Val/Val^ and BDNF^Val/Met^ mice, respectively. In conclusion, we report here that the BDNF Val66Met SNP knock‐in mice display an altered response to acute restraint stress in terms of hippocampal glutamate release, CREB phosphorylation, and neuronal activation, compared to wild‐type animals. Taken together, these results could partially explain the enhanced vulnerability to stressful events of Met carriers reported in both preclinical and clinical studies.

## Graphical abstract

Heterozygous brain‐derived neurotrophic factor (BDNF) Val66Met knock‐in mice display a higher response to acute restraint stress in terms of glutamate release and c‐fos gene expression. This was paralleled by different and selective phosphorylation of synapsin I at Ser^9^ and Ser^603^ in BDNF^Val/Val^ and BDNF^Val/Met^ mice respectively.

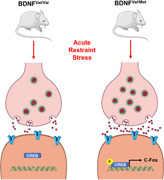

## 1 INTRODUCTION

Stress is a physiological response to challenging or threatening environments that stimulates body adaptation and increases the chances of survival (McEwen, 2007). The physiological response to acute stress implies the rapid activation of the hypothalamic–pituitary–adrenal (HPA) axis, leading to the release in the bloodstream of glucocorticoids, and corticosterone in rodents (de Kloet et al., 1998; Mourtzi et al., 2021). Nevertheless, stressful life events impact memory and cognition and are considered the major environmental risk factors in the onset of neuropsychiatric disorders, while alterations of HPA axis activity have been reported in depressed patients (Ceruso et al., 2020). However, the capability to cope with stress is diverse among individuals, which may depend on different factors such as age, gender, previous experiences, and genetic background (Cathomas et al., 2019; McEwen et al., 2015; Sanacora et al., 2022).

Brain‐derived neurotrophic factor (BDNF), the most abundant neurotrophin expressed in the adult brain, is one of the essential mediators of synapse formation, function, and plasticity in the mammalian brain and has been implicated in the pathophysiology of various stress‐related disorders as well as in the action of therapeutic drugs (Bramham & Messaoudi, 2005; Edelmann et al., 2014). Accordingly, several preclinical animal models have clearly shown that stress reduces BDNF expression, especially in the hippocampus (HPC) and prefrontal cortex, while both pharmacological and environmental therapeutical approaches can enhance BDNF expression in these same regions (Björkholm & Monteggia, 2016; Castrén & Monteggia, 2021; Nestler et al., 2002; Notaras & van den Buuse, 2020).

A functional single‐nucleotide polymorphism (SNP) has been identified in the human BDNF gene, which leads to the substitution of a valine with a methionine at the codon 66 (BDNF Val66Met; rs6265). This SNP is common in humans, with an allele frequency of 20%–30% in Caucasians, and is recognized as a contributing factor in morphological/functional brain variability (Miranda et al., 2019; Notaras et al., 2015; Tsai, 2018). The BDNF Val66Met SNP reduces the expression and the activity‐dependent release of BDNF and has been correlated in humans with a smaller hippocampal volume, memory impairments, increased predisposition to develop neuropsychiatric and neurodegenerative disorders, and altered vulnerability to stress (Chen et al., 2006; Egan et al., 2003; Hosang et al., 2014). In line with this evidence, BDNF^Met/Met^ knock‐in mice show a reduction in hippocampal volume, deficits in hippocampal‐dependent memory, and an anxious‐like phenotype (Chen et al., 2006). BDNF^Val/Met^ mice also have alterations in hippocampal gene expression involved in dendritic and spine remodeling (Mallei et al., 2018), reduced response to the beneficial effects of antidepressants and physical exercise (Bath et al., 2012; Ieraci et al., 2020), and enhanced susceptibility to stress exposure (Sandrini et al., 2018; Yu et al., 2012). However, the mechanisms underlying the enhanced vulnerability to stress is still largely unknown.

Alteration of the glutamatergic system is considered a key mechanism of stress‐related neuropsychiatric disorders (Duman et al., 2019; Sanacora et al., 2012, 2022). Clinical studies have consistently reported in patients with mood and anxiety disorders a volume reduction in brain areas enriched with glutamatergic neurons, such as the HPC and prefrontal cortex (Belleau et al., 2019). Consistently, several studies in animal models of depression have shown that stress promotes dendritic atrophy, a decrease in synapse number, and altered connectivity in the HPC and prefrontal cortex (McEwen et al., 2016; Musazzi et al., 2015; Woo et al., 2021). Moreover, acute stress or corticosterone administration enhances the glutamate release in the HPC and prefrontal cortex of rodents (Popoli et al., 2012; Sanacora et al., 2012). Interestingly, BDNF is critical for the maturation of glutamatergic neurons, regulates glutamate release, and influences excitatory synapse formation (Carvalho et al., 2008), while the BDNF Val66Met SNP impairs glutamatergic transmission and synaptic plasticity in the mouse HPC (Ninan et al., 2010). However, it is not known if the BDNF Val66Met SNP may regulate the release of glutamate induced by acute stress. This work is aimed at investigating whether the presence of the BDNF Val66Met SNP can influence the presynaptic glutamate release induced by acute restraint stress in the HPC of mice and to studying possible related molecular mechanisms and changes.

## 2 MATERIALS AND METHODS

### 2.1 Animals

Male BDNF^Val/Val^ and BDNF^Val/Met^ mice (3–4 months old) were used in the study (Chen et al., 2006). Mice were housed under standard conditions (20°C–22°C, 12h light/dark cycle, light on at 7 a.m.), with water and food ad libitum. Animal handling and experimental procedures were performed in accordance with the European Community Council Directive 2010/63/UE and approved by the Italian legislation on animal experimentation (Decreto Legislativo 26/2014, authorization N 349/2015‐PR). All efforts were made to minimize animal distress and to reduce the number of animals used in this study.

### 2.2 Restraint stress protocol

BDNF^Val/Val^ and BDNF^Val/Met^ mice were randomly divided into control (CNT) and acute restraint stress groups. For acute restraint stress, mice were individually restrained for 30 min in well‐ventilated 50‐ml polypropylene centrifuged tube (Ieraci et al., 2015). Acute restraint stress was performed during the morning period. CNT mice were left undisturbed in their home cages. Animals were killed by decapitation immediately after acute restraint stress protocol. The HPC was dissected on ice and right/left areas were pooled for purification of synaptosomes for glutamate release experiments, or randomly assigned to the purification of subcellular fractions for Western blot analyses or RNA extractions. Glutamate release and biochemistry/molecular analyses were conducted blinded.

### 2.3 Corticosterone serum levels measurement

For plasma preparation, trunk blood was collected on an ice‐cooled tube containing Ethylenediaminetetraacetic acid 0.5 M pH 8.00, separated by centrifugation, and stored at −80°C. Plasma corticosterone levels were measured using a commercial kit (Corticosterone ELISA kit; Enzo Life Sciences) (Ieraci et al., 2015).

### 2.4 Preparation of purified synaptosomes and neurotransmitter release experiments

Purification and superfusion of synaptic terminals (synaptosomes) were performed as previously reported (Musazzi et al., 2017). Synaptosomes were freshly prepared from homogenized HPC (homogenization buffer: 0.32 M sucrose, buffered at pH 7.4 with Tris‐HCl) of 8–12 animals/group, by centrifugation, and incubated at 37°C for 15 min in standard physiological medium (140 mM NaCl, 3 mM KCl, 1.2 mM MgSO_4_, 1.2 mM CaCl_2_, 1.2 mM NaH_2_PO_4_, 5 mM NaHCO_3_, 10 mM glucose, 10 mM HEPES, pH 7.4), in the presence of 0.05 μM [3H]d‐Aspartate ([3H]d‐Asp; Perkin Elmer Italia), a nonmetabolizable analog of glutamate used to label the synaptosomal glutamate releasing pools.

Aliquots of the synaptosomal suspension (about 100 μg) were distributed on microporous filters placed at the bottom of a set of parallel superfusion chambers maintained at 37°C (Superfusion System; Ugo Basile). Superfusion was then started with a standard medium at a rate of 0.5 ml/min and continued for 48 min. After 36 min of superfusion, samples were collected as follows: two 3‐min samples (*t* = 36–39 and 45–48 min; basal release) before and after one 6‐min sample (*t* = 39–45 min; stimulus‐evoked release). Stimulation with a 90 s pulse of 15 mM KCl was applied at *t* = 39 min. Radioactivity was determined in each sample collected and in the superfused filters by liquid scintillation (Ultima Gold, Perkin Elmer). Tritium released in each sample was calculated as a fractional rate × 100 (percentage of the total synaptosomal neurotransmitter content at the beginning of the respective sample collection). The stimulus‐evoked neurotransmitter overflow was estimated by subtracting the transmitter content of the two 3‐min fractions representing the basal release from that in the 6‐min fraction collected during and after the stimulating pulse. Radioactivity was determined in each sample collected and in the superfused filters by liquid scintillation counting. Tritium released in each sample was calculated as a fractional rate × 100 (percentage of the total synaptosomal neurotransmitter content at the beginning of the respective sample collection).

### 2.5 Preparation of subcellular fractions and western blot analysis

HPC was homogenized 1:10 (w/v) by a loose‐fitting Potter in homogenization buffer (0.28 M sucrose buffered at pH 7.4 with Tris, containing phosphatase inhibitors (Thermo‐Fisher Scientific) and 2 ml/ml of protease inhibitor cocktail [Sigma‐Aldrich]). Total homogenates were centrifuged for 5 min at 1,000*g*, and the resulting pellets, enriched in nuclei (P1), were resuspended in lysis buffer (120 mM NaCl, 20 mM HEPES pH 7.4, 0.1 mM EGTA, 0.1 mM DTT, protease and phosphatase inhibitors as in HB). Synaptosomes were prepared by centrifugation as above and synaptic membrane fraction was prepared by centrifugation as described previously (Musazzi et al., 2010; Treccani et al., 2014).

Western blot analysis was carried out by incubating polyvinylidene difluoride (PVDF) membranes containing electrophoresed proteins from nuclear fractions with antibodies for mineralocorticoid receptor (MR) (Santa Cruz Biotechnology), glucocorticoid receptor (GR) 1:500 (Santa Cruz Biotechnology), phospho‐Ser^232^ GR 1:1000 (Cell Signalling), cAMP‐response element binding protein (CREB) 1:1,000 (Cell Signalling), phospho‐Ser^133^ CREB 1:2,000 (Cell Signalling), and β‐actin 1:10,000 (Merck Group). On the other hand, PVDF membranes containing electrophoresed proteins from presynaptic membranes were incubated with monoclonal antibodies for synapsin I 1:2000 (Synaptic System) and β‐actin 1:20,000 (Sigma‐Aldrich), and polyclonal antibodies for P‐synapsin I Ser^9^ 1:1,000; P‐synapsin I Ser^306^ 1:1,000 (Cell Signaling) (Ieraci & Herrera, 2018). Following incubation with appropriated fluorochrome‐ (Li‐Cor Biotechnology) or peroxidase‐coupled (Merck Group) secondary antibodies, protein bands were detected by using Odyssey (Li‐Cor Biotechnology) or ECL (BioRad Laboratories S.r.l.) respectively. Total expression was normalized for β‐actin levels in the same membrane, while phosphorylation levels were normalized for the relative total protein. Standardization and quantitation were performed with ImageStudio (Li‐Cor Biotechnology) or Quantity One (BioRad Laboratories S.r.l.) software.

### 2.6 RNA isolation and reverse transcription

RNA isolation and reverse transcription were performed as previously described (Ieraci, Barbieri, et al., 2020). Briefly, total RNA from the HPC was extracted using the Direct‐zol RNA MiniPrep (Zymo Research, purchased by Euroclone) according to the manufacturer's instructions and quantified by absorption at 260 nm measured by UV spectrophotometry (NanoVue, GE Healthcare Europe GmbH). Complementary DNA was synthetized using the iScript kit (Bio‐Rad) according to the manufacturer's instructions.

### 2.7 Quantitative real‐time polymerase chain reaction (qPCR)

qPCR analysis was performed on a 7900HT Fast PCR System (Applied Biosystems by Life Technologies Italia) using iTaq Universal SYBR Green Supermix (Bio‐Rad).

The primers used were as follows: *c‐Fos*: forward 5′‐CTGCAGCCAAGTGCCGGAATC‐3′, reverse 5′‐GGCAATCTCAGTCTGCAACGC‐3′; *Arc*: forward 5′‐AGCCCAAACTCAAGCGCTTT‐3′, reverse 5′‐GAAGGCTCAGCTGCCTGCCTC‐3; *Bdnf*: forward 5′‐TCGTTCCTTTCGAGTTAGCC‐3′, reverse 5′‐TTGGTAAACGGCACAAAAC‐3; *Gapdh*: forward 5′‐CGTGCCGCCTGGAGAAACC‐3′, reverse 5′‐ TGGAAGAGTGGGAGTTGCTGTTG‐3′; *Rps18*: forward 5′‐TGGAGCGAGTGATCACCATCA‐3′, reverse 5′‐CCTCACGCAGCTTGTTGTCTA‐3′. PCR cycling conditions were: 10 min at 95°C, 40 cycles of 15 s at 95°C and 1 min at 60°C. Data from qPCR were normalized on the mean of two reference genes (*Rps18* and *Gapdh*). Analysis of the melting curve verified the specificity of the PCR products. Relative amounts were determined using the comparative Cq method (Mallei et al., 2019).

### 2.8 Statistical analysis

Statistical analysis of the data was carried out using GraphPad Prism6 (GraphPad Software Inc.). Results are presented as mean ± standard error of the mean (SEM). Normal distributions were verified by the Kolmogorov–Smirnov's test. For normally distributed data, statistical analyses were performed by a two‐way analysis of variance (ANOVA) (genotype × stress) followed by the Newman–Keuls posthoc multiple comparison test. For non‐normally distributed data, statistical analyses were performed by the Kruskal–Wallis test followed by the Dunn's multiple comparison test.

## 3 RESULTS

### 3.1 Acute restraint stress activated the hypothalamus–pituitary–adrenal axis in both BDNF^Val/Val^ and BDNF^Val/Met^ mice

To assess the HPA axis reactivity and the consequent activation of corticosterone receptors, we measured plasma corticosterone levels, as well as the nuclear levels of MR, GR, and phospho‐Ser^232^ GR (pGR) as an indirect measure of receptor activation (Mifsud & Reul, 2018).

**Figure 1:**
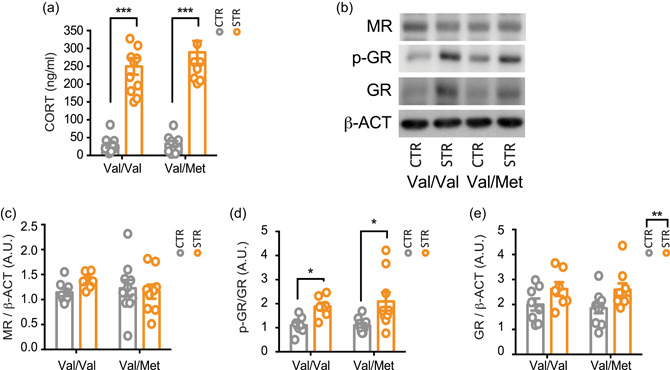
Acute restraint stress activated the hypothalamus–pituitary–adrenal axis in both BDNF^Val/Val^ and BDNF^Val/Met^ mice. (a) Corticosterone plasma levels were measured immediately after the 30 min of acute restraint stress. Data are reported as mean ± SEM (*n* = 9–11 mice/group). Kruskal–Wallis test followed by the Dunn's multiple comparison test. ****p < 0.0001. (b–e) Western blot analysis of the hippocampal nuclear fraction of total MR (c), phosphorylated GR (p‐GR) (d) and total GR (e). (b) Representative western blot images of total MR, total GR, p‐GR, and β‐actin. Densitometric analysis was obtained as a ratio of MR/β‐actin (c), p‐GR/GR (d) and GR/β‐actin (e). Data are reported as mean ± SEM (*n* = 7–10 mice/group). Kruskal–Wallis test followed by the Dunn's multiple comparison test (a, d) and two‐way ANOVA followed by Newman–Keuls multiple comparison test (c, e). *p < 0.05; ***p < 0.001. ANOVA, analysis of variance; A.U., arbitrary unit; BDNF, brain‐derived neurotrophic factor; GR, glucocorticoid receptor; MR, mineralocorticoid receptor; SEM, standard error of the mean.

Kruskal–Wallis test (*p* < 0.0001) followed by Dunn's multiple comparison test revealed that acute restraint stress induced a similar increase of plasma corticosterone levels in both BDNF^Val/Val^ (*p* < 0.001) and heterozygous BDNF^Val/Met^ mice (*p* < 0.001) (Figure 1a). Moreover, we found no significant difference among the experimental groups in the nuclear expression of MR (two‐way ANOVA; stress: *F*_(1, 30)_ = 1.71; *p* = 0.35; genotype: *F*_(1, 30)_ = 2.16; *p* = 0.403; interaction: *F*_(1, 30)_ = 6.133; *p* = 0.164) (Figure 1b,c), while Kruskal–Wallis test (*p* = 0.0016) followed by Dunn's multiple comparison test showed that stress similarly increases pGR levels in both the genotypes (*p* < 0.05). Moreover, two‐way ANOVA revealed a significant effect of stress for total GR (*F*_(1, 30)_ = 20.43; *p* = 0.0089), with no effect of genotype (*F*_(1, 30)_ = 0.1250; *p* = 0.7262) or stress × genotype interaction (*F*_(1, 30)_ = 0.0615; *p* = 0.8058) (Figure 1b,d,e). These results suggest that acute restraint stress induces similar activation of the HPA axis in both genotypes.

### 3.2 Acute restraint stress increased depolarization‐evoked glutamate release in the HPC of BDNF^Val/Met^

**Figure 2:**
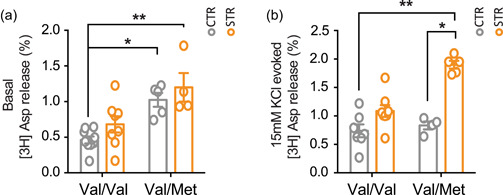
Acute restraint stress induced a higher increase of presynaptic glutamate release in the hippocampus of BDNF^Val/Met^ than in BDNF^Val/Val^ mice. (a) Basal glutamate release from hippocampal synaptosomes in superfusion. Data are reported as mean ± SEM (*n* = 4–9 mice/group). Kruskal–Wallis test followed by Dunn's multiple comparison test. ***p < 0.001. (b) 15 mM KCl‐evoked glutamate release from hippocampal synaptosomes in superfusion. The net depolarization‐evoked overflow was calculated by subtracting transmitter content of the basal outflow. Data are reported as mean ± SEM (*n* = 4–8 mice/group). Kruskal–Wallis test followed by Dunn's multiple comparison test. *p < 0.01. BDNF, brain‐derived neurotrophic factor; SEM, standard error of the mean. % = % overflow.

To assess whether the BDNF Val66Met polymorphism may alter presynaptic glutamate release in the HPC and/or interfere with the glutamatergic changes induced by acute stress, we measured both basal and depolarization‐evoked release of glutamate from hippocampal synaptosomes in superfusion. Kruskal–Wallis test (*p* = 0.0021) followed by Dunn's multiple comparison test revealed that basal glutamate release was higher in BDNF^Val/Met^, independently of stress exposure, compared to BDNF^Val/Val^ mice (*p* < 0.05) (Figure 2a). Moreover, as regards depolarization‐evoked glutamate release, we found that acute restraint stress increased the depolarization‐evoked release of glutamate only in BDNF^Val/Met^ (Kruskal–Wallis test: *p* = 0.0016; Dunn's post hoc test: *p* < 0.05) but not in BDNF^Val/Val^ mice (Figure 2b).

### 3.3 Acute restraint stress induced different changes of synapsin I phosphorylation in the HPC of wild‐type and BDNF^Val/Met^ mice

**Figure 3:**
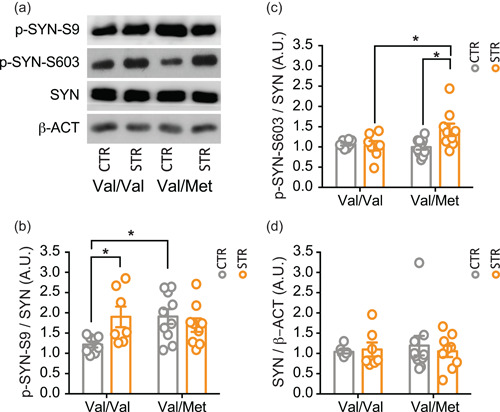
Acute restraint stress induced selective changes in synapsin I phosphorylation in the hippocampus of BDNF^Val/Val^ and BDNF^Val/Met^ mice. (a–c) Western blot analysis of total synapsin I (b), phospho‐ser^9^ synapsin I (c), phospho‐ser^603^ synapsin I (d) in hippocampal presynaptic membranes. (a) Representative western blot images of total SynI, p‐ser^9^‐SynI, p‐ser^603^‐SynI, and β‐actin. Densitometric analysis was obtained as a ratio of SynI/β‐actin (b), p‐ser^9^‐SynI/SynI (c), p‐ser^603^‐SynI/SynI (d). Data are reported as mean ± SEM (*n* = 7–10 mice/group). Two‐way ANOVA followed by Newman–Keuls multiple comparison test (b and c) and Kruskal–Wallis test followed by the Dunn's multiple comparison test (d). *p < 0.05. ANOVA, analysis of variance; A.U., arbitrary unit; BDNF, brain‐derived neurotrophic factor; SEM, standard error of the mean.

To identify possible mechanisms involved in the changes of glutamate release induced by acute restraint stress in BDNF^Val/Val^ and BDNF^Val/Met^ mice, we analyzed the expression and phosphorylation levels in presynaptic membranes of synapsin I, which is well‐known for playing a key role in synaptic vesicle trafficking (Song & Augustine, 2015) and has been previously shown to take part in the molecular processes involved in the increase of glutamate release induced by acute stress (Treccani et al., 2014). Concerning synapsin I phosphorylation at both Ser^9^ and Ser^603^, we found a significant effect of stress × genotype interaction (Ser^9^: *F*_(1, 30)_ = 6.172; *p* = 0.019; Ser^603^: *F*_(1, 30)_ = 5.002; *p* = 0.033), but not of genotype (Ser^9^: *F*_(1, 30)_ = 1.809; *p* = 0.189; Ser^603^: *F*_(1, 30)_ = 2.43; *p* = 0.129) or stress (Ser^9^: *F*_(1, 30)_ = 1.733; *p* = 0.0.198; Ser^603^: *F*_(1, 30)_ = 3.468; *p* = 0.072) (Figure 3a,b,c). Post hoc analysis revealed that acute restraint stress selectively increased the phosphorylation levels of synapsin I at Ser^9^ in BDNF^Val/Val^ (*p* < 0.05) but not in BDNF^Val/Met^ mice, in which however phosphorylation levels at Ser^9^ were higher in control compared to control BDNF^Val/Val^ mice (*p* < 0.05) and were not further increased by stress (Figure 3a,b). Instead, synapsin I phosphorylation at Ser^603^ was unchanged between the two genotypes in basal conditions, but acute restraint stress significantly increased its levels only in BDNF^Val/Met^ (*p* < 0.05) and not in BDNF^Val/Val^ mice (Figure 3a,c).

Moreover, total synapsin I levels were similar in all experimental groups (genotype: F_(1, 30)_ = 0.039; *p* = 0.845; stress: F_(1, 30)_ = 0.051; *p* = 0.823; stress × genotype interaction: F_(1, 30)_ = 0.898; *p* = 0.351) (Figure 3a,d).

### 3.4 Acute restraint stress induced a higher increase of CREB phosphorylation in the HPC of BDNF^Val/Met^ compared to BDNF^Val/Val^ mice

Having found selective glutamatergic alterations induced by acute restraint stress in the two genotypes, we next assessed whether this was accompanied by changes in intracellular signaling. In particular, we analyzed the nuclear expression and phosphorylation levels of the transcription factor CREB, one of the major regulators of neurotrophic responses, which is also known to be activated by glutamatergic stimulation and after acute stress (Ieraci et al., 2015; Mao et al., 2004; Nasca et al., 2017).

**Figure 4:**
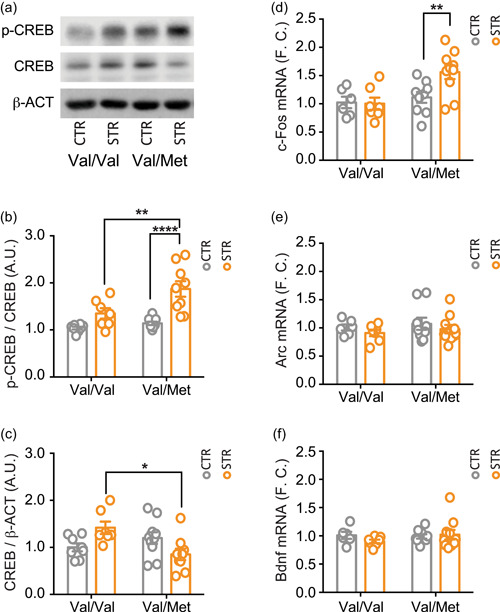
Acute restraint stress increased CREB phosphorylation and cFOS mostly in the hippocampus of BDNF^Val/Met^ mice. (a–c) Western blot analysis of hippocampal nuclear fraction of phosphorylated CREB (b) and total CREB (c). (a) Representative western blot images of CREB, p‐CREB, and β‐actin. Densitometric analysis were obtained as ratio of p‐CREB/CREB (b) and CREB/β‐actin (c). Data are reported as mean ± SEM (*n* = 7–10 mice/group). Two‐way ANOVA followed by Newman–Keuls multiple comparison test (b, d–f) and Kruskal–Wallis test followed by the Dunn's multiple comparison test (c). *p < 0.05; **p < 0.01; ****p < 0.0001. (d–f) qPCR analysis of hippocampal mRNA levels of *c‐Fos* (d), *Arc* (e) and *Bdnf* (f). Data are reported as mean ± SEM (*n* = 6–10 mice/group). One‐way ANOVA followed by Newman–Keuls multiple comparison test. **p < 0.01. ANOVA, analysis of variance; A.U., arbitrary unit; BDNF, brain‐derived neurotrophic factor; CREB, cAMP‐response element binding protein; F.C.; fold change; mRNA, messenger RNA; qPCR, quantitative real‐time PCR; SEM, standard error of the mean.

For phospho‐Ser^133^ CREB levels, we found significant effects of genotype (F_(1, 31)_ = 9.692; *p* = 0.040), stress (F_(1, 31)_ = 28.04; *p* < 0.0001), and stress × genotype interaction (F_(1, 31)_ = 4.352; *p* = 0.0453). Post hoc analysis showed that acute restraint stress significantly increased CREB phosphorylation exclusively in BDNF^Val/Met^ mice but not in BDNF^Val/Val^ (BDNF^Val/Met^ + RS vs. BDNF^Val/Met^ *p* < 0.0001; BDNF^Val/Met^ + RS vs. BDNF^Val/Val^ + RS *p* < 0.001; BDNF^Val/Met^ + RS vs. BDNF^Val/Val^ *p* < 0.0001) (Figure 4a,b). Differently, regarding total CREB levels, two‐way ANOVA showed only a significant effect of the stress × genotype interaction (F_(1, 31)_ = 11.46; *p* = 0.0019), with no effects of genotype (F_(1, 31)_ = 2.798; *p* = 0.1044) or stress (F_(1, 31)_ = 0.1349; *p* = 0.7159) (Figure 4a,c). CREB levels were lower in stressed BDNF^Val/Met^ mice compared to stressed BDNF^Val/Val^ mice (*p* < 0.05) (Figure 4a,c).

### 3.5 Acute restraint stress increased the expression of c‐fos only in BDNF^Val/Met^ mice

Finally, we analyzed whether the differential activation of CREB induced by acute restraint stress in BDNF^Val/Val^ and BDNF^Val/Met^ mice correlated with transcriptional changes of selected downstream genes, such as *c‐Fos*, *Arc*, and *Bdnf*. ANOVA analysis of *c‐Fos* messenger RNA (mRNA) levels, measured by quantitative PCR, showed significant effects of genotype (F_(1, 28)_ = 8.069; *p* = 0.0083) and stress × genotype interaction (F_(1, 28)_ = 4.26; *p* = 0.0484), but no effect of stress (F_(1, 28)_ = 3.48; *p* = 0.0726) (Figure 4d). As for CREB phosphorylation, acute stress significantly increased *c‐Fos* expression levels exclusively in BDNF^Val/Met^ mice (BDNF^Val/Met^ + RS vs. BDNF^Val/Met^ *p* < 0.01; BDNF^Val/Met^ + RS vs. BDNF^Val/Val^ + RS *p* < 0.01; BDNF^Val/Met^ + RS vs. BDNF^Val/Val^ *p* < 0.01) (Figure 4d). In contrast, no significant differences among experimental groups were found for *Arc* and *Bdnf* mRNA levels (*Arc*: genotype F_(1, 28)_ = 0.615; *p* = 0.439; stress F_(1, 28)_ = 1.257; *p* = 0.272; stress × genotype interaction F_(1, 28)_ = 0.00038; *p* = 0.985. *Bdnf*: genotype F_(1, 28)_ = 0.557; *p* = 0.462; stress F_(1, 28)_ = 0.318; *p* = 0.578; stress × genotype interaction F_(1, 28)_ = 1.18; *p* = 0.299) (Figure 4e,f). Overall, these results are in line with a higher stimulation of glutamatergic transmission in the HPC of BDNF^Val/Met^ mice compared to BDNF^Val/Val^ mice.

## 4 DISCUSSION

In this study, we showed that acute restraint stress, although leading to a similar activation of the HPA axis, increased depolarization‐evoked presynaptic release of glutamate, phosphorylation of CREB, and levels of the immediate early gene *c‐Fos* in the HPC of BDNF^Val/Met^ compared to BFNF^Val/Val^ mice. Moreover, acute restraint stress selectively increased the phosphorylation levels of synapsin I at Ser^9^ and at Ser^603^ in BFNF^Val/Val^ and BDNF^Val/Met^ mice, respectively.

Activation of the HPA axis represents a primary hormonal response to a homeostatic challenge. Temporal activation of the HPA axis depends on stress duration and type but, being essential for coping with the environment and increase of survival probability, the stress response is extremely fast in nature (Herman et al., 2016). Acute stress efficiently drives HPA stress response, and feedback mechanisms effectively terminate the response after the stressor subsides.

The overall effect of acute restraint stress on the HPA axis is well documented: corticotropin‐releasing hormone, adrenocorticotropic hormone, and glucocorticoid secretion all increase significantly during the stressor period but returned to basal levels within an hour after stress. Previous evidence showed that corticosterone levels are remarkably increased after 30 min of restraint stress but rapidly go back to control levels within 120 min (Mcclennen et al., 1998).

Interestingly, it has also been reported that plasma corticosterone levels peaked 30 min after a single saline injection, a very mild acute stress and that this increase was higher in BDNF^+/‐^ heterozygous mice compared to wild‐type mice (Advani et al., 2009). This and other evidence suggest that BDNF may be involved in the regulation of the HPA axis response to stress (Naert et al., 2015; Numakawa et al., 2013). However, the possible role of the BDNF Val66Met SNP in the HPA reactivity and cortisol release after stress exposure is still controversial. While some human studies have reported a stronger cortisol response in BDNF Met/Met compared to Val/Val carriers (Schüle et al., 2006), others have found a greater rise in Val/Val compared to Met carriers (Alexander et al., 2010), or similar cortisol release among different genotypes (Hennings et al., 2019). Here, by using the knock‐in BDNF Val66Met mice in a well‐controlled laboratory environment, we found that acute restraint stress similarly rises corticosterone plasma levels in both BDNF^Val/Val^ and BDNF^Val/Met^ male mice. Interestingly, it was previously reported that, after seven consecutive days of restraint stress, the corticosterone release was greater in BDNF^Val/Met^ compared to BDNF^Val/Val^ mice (Yu et al., 2012). This may suggest that a single stressful event can induce a similar release of corticosterone in both BDNF^Val/Val^ and BDNF^Val/Met^ mice, while repeated sessions can modify HPA response. Further studies will be needed to better clarify whether this effect could be due either to a sensitization of the HPA axis in BDNF^Val/Met^ mice or to a lower activation in BDNF^Val/Val^ mice.

The comparable increases of nuclear GR and pGR levels following acute restraint stress in both BDNF^Val/Val^ and BDNF^Val/Met^ mice are consistent with the similar corticosterone rise. These results are in line with earlier data showing an increase in phospho‐Ser^232^ GR following acute and chronic stress (Adzic et al., 2009).

It has been reported that BDNF Val66Met SNP impairs NMDA receptor‐dependent synaptic transmission (Ninan et al., 2010) and affects the stress response in the HPC (Notaras et al., 2016; Yu et al., 2012). However, to the best of our knowledge, here we demonstrated for the first time that the BDNF Val66Met SNP leads to alterations in presynaptic glutamate release induced by acute stress in the HPC. Interestingly, in BDNF^Val/Met^ mice basal glutamate release was higher compared to BDNF^Val/Val^ and acute stress induced a remarkable enhancement of depolarization‐evoked glutamate release in BDNF^Val/Met^ mice. Overall, these results are suggestive of presynaptic glutamate abnormalities in the HPC of BDNF^Val/Met^ mice. Basal glutamate release has been associated with synaptic homeostasis, a process stabilizing neuronal and circuit activity (Turrigiano, 2012). An impairment of these mechanisms is likely to be involved in maladaptive stress response and increased psychopathological risk, while homeostatic synaptic plasticity has been implicated in antidepressant effects (Kavalali & Monteggia, 2020). Similarly, an abnormal glutamatergic activation under stress conditions as observed for BDNF^Val/Met^ mice could induce noxious effects and alter physiological glutamate transmission and plasticity (Musazzi et al., 2011; Popoli et al., 2012; Sanacora et al., 2022). Accordingly, the BDNF Val66Met SNP has been associated with impairments in hippocampal glutamatergic transmission (Ninan et al., 2010).

Acute stressors stimulate the release of several neurotransmitters (glutamate, GABA, acetylcholine, dopamine) in different brains regions (HPC, prefrontal cortex, amygdala, nucleus accumbes), that are related to the activation and modulation of behavioral processes to cope with the stress (Bhakta et al., 2017; Mora et al., 2012). Here we have specifically shown that the BDNF Val66Met SNP alters the release of glutamate in the HPC. However, we can't exclude that the release of other neurotransmitters could be differently affected in distinctive brain regions, besides glutamate in the HPC, in BDNF Val66Met carrier subjects.

Previous evidence has established that synapsin I regulates glutamate release and is involved in mechanisms increasing glutamate release under acute stress conditions (Musazzi et al., 2017; Nichols et al., 1992; Treccani et al., 2014). Unphosphorylated synapsin I binds to actin synaptic vesicles, thus confining them to the reserve pool. Under depolarizing conditions, synapsin I is phosphorylated at different sites by several protein kinases, leading to release of presynaptic vesicles from actin filaments and mobilization towards the ready releasable pool (RRP) of vesicles (Hackett & Ueda, 2015; Musazzi et al., 2017; Revest et al., 2010). We have previously shown that the selective phosphorylation of synapsin I at Ser^9^ is required for the mobilization of vesicles toward the RRP and increase of presynaptic glutamate release induced by acute stress (Musazzi et al., 2017; Treccani et al., 2014). In line with this evidence, we observed here that synapsin I phosphorylation levels at Ser^9^ were basally high in unstressed BDNF^Val/Met^ mice and were not further modified by acute restraint stress, while acute stress increased Ser^603^ phosphorylation. These results suggest that the mechanisms of synapsin I regulation by phosphorylation is dysregulated in BDNF^Val/Met^ mice, to the point that Ser^9^ phosphorylation is not further modified by acute stress. On the other hand, phosphorylation of synapsin I at Ser^603^, which is not affected in wild‐type animals, might play a role in the excessive increase of glutamate release induced by acute stress in BDNF^Val/Met^ mice. Although the involvement of phosphorylation at other sites cannot be excluded, since Ser^603^ is phosphorylated by calcium‐calmodulin‐dependent protein kinase II in response to calcium influx during nerve terminal activation (Huttner & Greengard, 1979), this result is in line with alteration in presynaptic calcium homeostasis in BDNF^Val/Met^ mice. This in turn might lead to hyperexcitability and excitotoxic risk, in line with the higher susceptibility to behavioral consequences of chronic stress previously reported in BDNF^Val/Met^ mice compared with BDNF^Val/Val^ mice (Yu et al., 2012).

CREB is a nuclear transcription factor activated by phosphorylation, downstream of several intracellular cascades involved in neuronal activation and neuroplastic processes (Carlezon et al., 2005). Previous evidence consistently showed that acute stress increases CREB phosphorylation in the HPC (Alboni et al., 2011; Ieraci et al., 2015). Accordingly, we found that CREB phosphorylation was increased after acute restraint stress exposure in both BDNF^Val/Val^ and BDNF^Val/Met^ mice, although the increase was higher in BDNF^Val/Met^ compared to BDNF^Val/Val^ mice. This suggests that the observed presynaptic glutamatergic dysregulation in BDNF^Val/Met^ mice is accompanied by an abnormal cellular response. This hypothesis is confirmed by the expression of the immediate early gene *c‐Fos*, which is considered a good marker of neuronal activity (Benito & Barco, 2015). On the other hand, we showed that in the HPC of wild‐type BDNF^Val/Val^ mice acute restraint stress did not promote any significant change in the mRNA levels of *c‐Fos*. This result is consistent with previous work showing an increase of *c‐Fos* only 140 min after the end of 30 min acute restraint stress (Alboni et al., 2011). No changes were instead measured in *Arc* and *Bdnf* expression.

In conclusion, we found that knock‐in mice for the human BDNF Val66Met SNP show an abnormal response to acute restraint stress in terms of glutamate release, CREB phosphorylation, and neuronal activation. Taken together, these results could partially explain the increased susceptibility to stressful events of BDNF Met carriers evidenced in both preclinical and clinical studies. Future studies will be needed to better understand these mechanisms and to test whether there is a different short‐ and long‐term behavioral response to stress in BDNF^Val/Met^ mice compared to wild‐type BDNF^Val/Val^ mice.

## AUTHOR CONTRIBUTIONS

Alessandro Ieraci and Laura Musazzi designed the concept. Alessandro Ieraci, Laura Musazzi, Nathalie Sala, and Paolo Tornese performed the experiments. Alessandro Ieraci and Laura Musazzi analyzed the data. Alessandro Ieraci and Laura Musazzi prepared and wrote the original draft. Francis S. Lee and Maurizio Popoli critically revised the manuscript. All the authors read and approved the final manuscript.

## CONFLICT OF INTEREST

The authors declare no conflict of interest.
